# Young female with abdominal pain and intra‐abdominal free fluid: The risk of confirmation bias associated with point‐of‐care ultrasound

**DOI:** 10.1002/ajum.12320

**Published:** 2022-09-17

**Authors:** Laura Joyce, Jacques Loubser, Rex de Ryke, Alexandra McHaffie

**Affiliations:** ^1^ Department of Surgery University of Otago (Christchurch) Christchurch New Zealand; ^2^ Emergency Department Te Whatu Ora ‐ Waitaha Christchurch New Zealand; ^3^ Department of Radiology Te Whatu Ora ‐ Waitaha Christchurch New Zealand; ^4^ Department of Radiology University of Otago (Christchurch) Christchurch New Zealand

**Keywords:** bias, POCUS, point‐of‐care ultrasound

## Abstract

Confirmation bias is an ever‐present risk to the rapid decision‐making required in emergency departments (EDs). We present a case of a young woman who was brought to ED by ambulance with hypotension, syncope and vaginal bleeding, with a presumptive pre‐hospital diagnosis of ruptured ectopic pregnancy. On arrival in ED, she was found to have intra‐abdominal free fluid on bedside ultrasound. This finding could have been used by clinicians to confirm their suspicion of a ruptured ectopic; however, with further investigations, it was found that anaphylaxis was the most likely cause of the patient's symptoms. This case highlights that point‐of‐care ultrasound findings can play a potentially dangerous role in confirmation bias and that we should maintain an open mind when making a diagnosis by treating the patient, rather than the ultrasound picture.

## Introduction

Confirmation bias,^1^ the tendency to search for and interpret information in a way that confirms or supports one's prior beliefs, is constantly a risk to decision‐making in the emergency department (ED). ED diagnostic and management plans are often made with a paucity of information, in a high‐stress environment that requires rapid decision‐making. The diagnostic process often begins before the patient arrives in ED, with ambulance pre‐notifications creating a differential diagnosis in the clinician's mind. Diagnostic momentum can carry a prehospital presumptive diagnosis through the ED, where clinicians may search for evidence to confirm this diagnosis, while not considering other differentials. This is likely to be more common in high‐stake situations with unstable patients or those with potentially life‐threatening diagnoses, such as ruptured ectopic pregnancy, where rapid decision‐making is required.

## Case study

A 34‐year‐old woman with rheumatoid arthritis and polycystic ovarian syndrome was brought by ambulance to the ED with a history of acute onset abdominal pain, vaginal bleeding and syncope. She was also noted to have an urticarial rash. The ambulance pre‐notification was concerned for a possible ruptured ectopic pregnancy.

**Figure 1:**
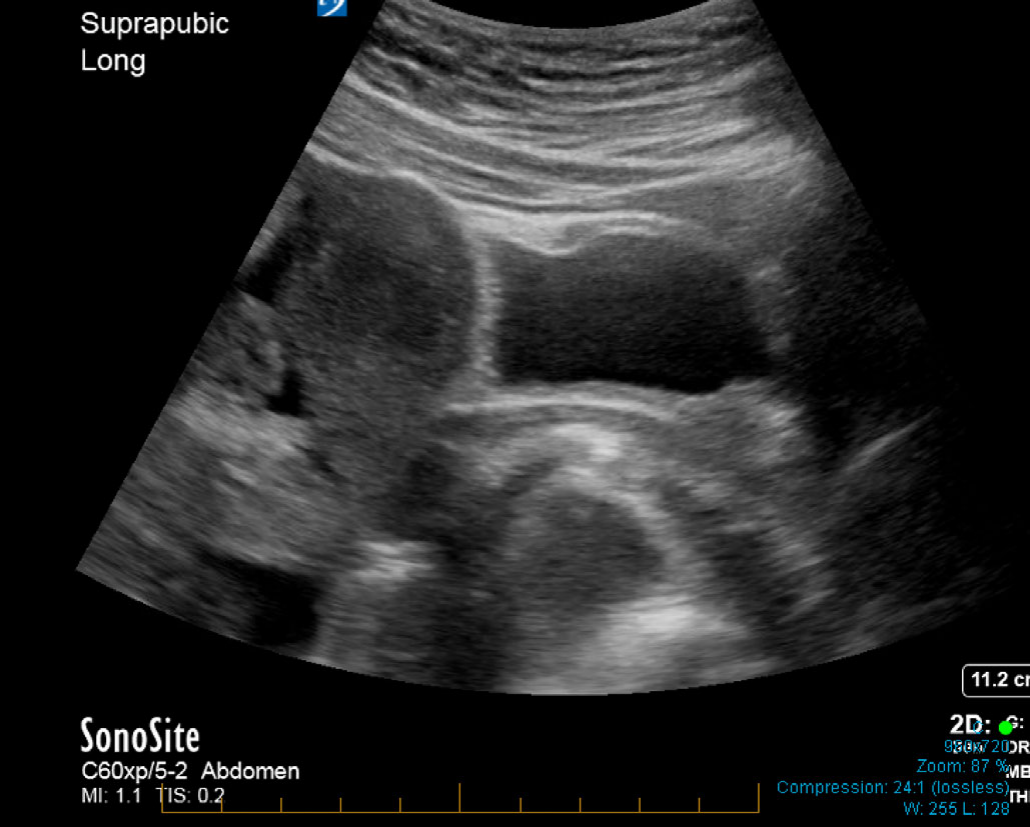
Suprapubic long view. [Colour figure can be viewed at wileyonlinelibrary.com]

**Figure 2:**
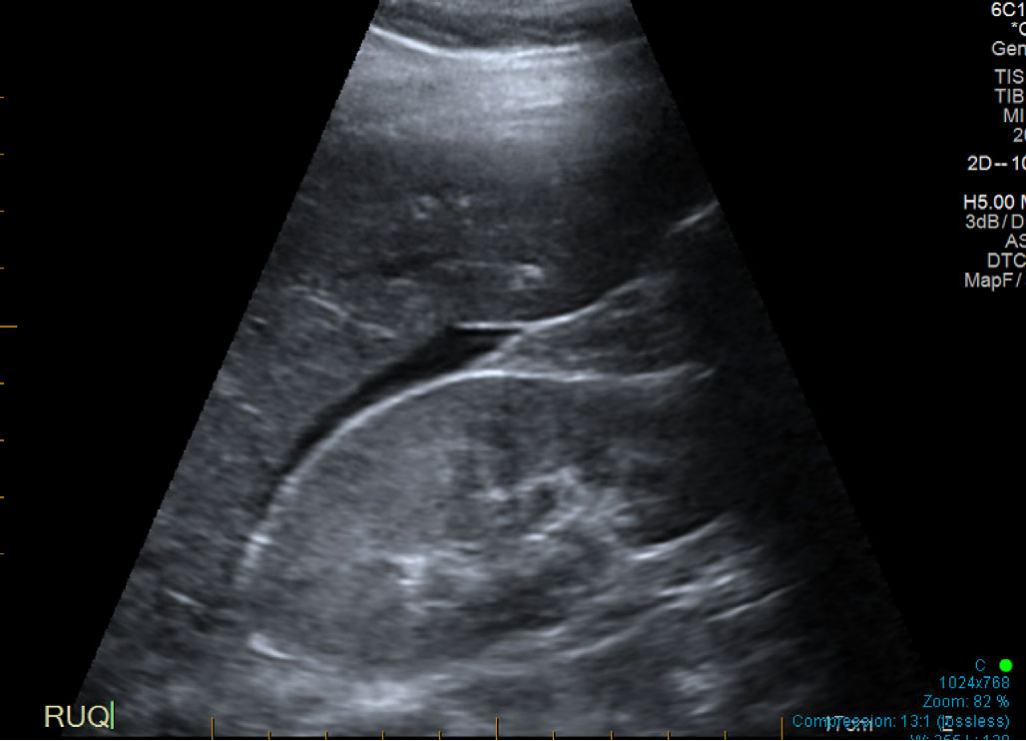
Right upper quadrant view. [Colour figure can be viewed at wileyonlinelibrary.com]

Pre‐hospital blood pressure was 65/45, and 700 mL of 0.9% saline iv was administered en route. On arrival in ED, her observations were GCS 15, BP 110/50, HR 78, RR 18 and oxygen saturation 98% on room air. The patient had an urticarial rash, but no facial swelling, wheeze or stridor. Her abdomen was diffusely tender, and point‐of‐care ultrasound (POCUS) undertaken by a ASUM Certificate in Clinician Performed Ultrasound (CCPU)‐qualified practitioner showed an empty uterus (Figure 1) with a moderate volume of intra‐abdominal free fluid (Figures 1 and 2).

Due to the history of syncope, pre‐arrival hypotension and urticarial rash, 0.5 mg of intramuscular adrenaline was administered in ED, with the resolution of the rash and improvement in observations. Urine pregnancy test was negative, although delayed by approximately 30 min due to the patient's inability to produce a urine sample, and a CT scan was performed to look for a cause of the pain, hypotension and intraperitoneal free fluid. CT abdomen showed a moderate volume of free fluid with relatively low Hounsfield units of 17 inconsistent with haemoperitoneum, with equivocal right iliac fossa small bowel thickening and enhancement.

Blood test (taken after adrenaline given) showed a normal haemoglobin level with a neutrophilia (WCC 33 and neutrophils 26) and normal U + Es/LFTs/amylase/CRP. The albumin level was normal at 32. Pregnancy beta‐HCG was <2. COVID PCR test was negative, tryptase was normal, and levels of protein checked with complement blood test the next day were also within normal limits. It is important to note that both urine and serum pregnancy test results can be delayed in ED due to patient's inability to produce a urine sample on demand and a lab turnaround time of 60–90 min.

The patient was reviewed by the gynaecology team who presumed that the vaginal bleeding was due to normal menstruation. The patient was admitted to general medicine overnight for observation. She was treated with prednisone and cetirizine and discharged the next day. She was subsequently reviewed in the clinic by both immunologist and rheumatologist, and retrospectively, her symptoms, and the free fluid, were thought to be due to anaphylaxis to ibuprofen that the patient had taken for presumed period pain.

## Discussion

Ectopic pregnancy is the leading cause for first‐trimester maternal mortality and may be present in up to 8% of ED presentations in early pregnancy.^2^ Emergency department physicians and paramedics are trained to diagnose ectopic pregnancy in any woman with abdominal pain and hypotension.^3^ POCUS is rapidly available at the bedside in most EDs, is accurate and may decrease the length of stay.^4^, ^5^

There is a paucity of literature describing the association between anaphylaxis and significant amounts of intra‐abdominal free fluid in humans. Angiotension‐converting enzyme (ACE) inhibitor–induced angioedema has been infrequently reported as a mimic of the ‘surgical abdomen’;^6^, ^7^ however, this patient was not taking an ACE inhibitor. Similarly, hereditary angioedema may rarely cause repeated episodes of acute abdominal pain with ascites.^8^ ‘Systemic capillary leak syndrome’, also known as Clarkson's disease, has been described as a rare disorder with repeated transient episodes of hypotensive shock and anasarca – including intra‐abdominal free fluid – characterised by acute paroxysmal capillary hyperpermeability.^9^, ^10^, ^11^ This syndrome would not usually be corrected by the administration of adrenaline, and therefore, anaphylaxis was felt to be a more likely diagnosis for this patient.

Point‐of‐care ultrasound performed by emergency department physician may rapidly identify intraperitoneal free fluid in suspected ectopic pregnancy, which predicts the need for operative management.^12^ However, the presence of intra‐abdominal free fluid in a young woman with abdominal pain and hypotension is not always indicative of ectopic pregnancy. In this case, there was a risk of confirmation bias where the presence of the urticarial rash could have been overlooked as it did not support the initial conclusion that a young woman with abdominal pain and hypotension has an ectopic pregnancy ‘until proven otherwise’. It was only the rash that made the clinician consider a differential of anaphylaxis and give adrenaline; however, this could have been missed without a full primary and secondary survey. The assumption that this patient had a ruptured ectopic pregnancy or ovarian cyst risked an unnecessary operation. Point‐of‐care ultrasound findings can play a role in confirmation bias – treat the patient, not the ultrasound picture.^13^

## Learning points

- Biases are a universal risk to clinical decision-making, and can be reduced by awareness.
- Complete your primary and secondary survey – if symptoms or signs are not congruent with your suspected diagnosis, slow down your thinking to avoid confirmation bias and consider alternative differentials.
- Anaphylaxis is a multi‐system disorder which may present in a variety of ways – intra‐abdominal free fluid can be a rare feature.

## Author contribution

The authors confirm contribution to the paper as follows: study conception and design: LJ. All authors reviewed the draft manuscript and approved the final version of the manuscript.

## Authorship statement

All authors acknowledge that (i) the authorship listing conforms with the journal's authorship policy and that (ii) all authors are in agreement with the content of the submitted manuscript.

## Funding

No funding information is provided.

## Conflicts of interest

None declared.

## Patient consent

Written consent was given by the patient involved in this case.

## Ethical approval

Ethics approval was not required by the host institution for a case study.
